# Identification of Three Novel PmGRI1 Genomic Resistance Islands and One Multidrug Resistant Hybrid Structure of Tn*7*-like Transposon and PmGRI1 in *Proteus mirabilis*

**DOI:** 10.3390/antibiotics10101268

**Published:** 2021-10-18

**Authors:** Boheng Ma, Xuechun Wang, Changwei Lei, Yizhi Tang, Juan He, Yufeng Gao, Yu Zhang, Hongning Wang

**Affiliations:** 1 College of Life Sciences, Sichuan University, Chengdu 610065, China; bohengm@163.com (B.M.); wangxuechuns@126.com (X.W.); leichangwei@126.com (C.L.); tangyizhi23@163.com (Y.T.); 2018222040040@stu.scu.edu.cn (J.H.); 2019222040103@stu.scu.edu.cn (Y.G.); yuzhang712@163.com (Y.Z.); 2 Key Laboratory of Bio-Resource and Eco-Environment of Ministry of Education, Sichuan University, Chengdu 610065, China; 3 Animal Disease Prevention and Food Safety Key Laboratory of Sichuan Province, Sichuan University, Chengdu 610065, China

**Keywords:** *Proteus mirabilis*, antibiotic resistance, genomic island, PmGRI1, Tn*7*

## Abstract

The widespread use of antibiotics in large-scale livestock production has led to serious antibiotic resistance. *Proteus mirabilis* is an important pathogenic bacterium on large-scale farms. Chromosomally localized mobilizable genetic elements (genomic islands) and mobile genetic elements (Tn*7*-like transposons) play an important role in the acquisition and transmission of resistance genes by *P. mirabilis*. To study the prevalence and resistance characteristics of antibiotic-resistant genomic islands in *P. mirabilis* of animal origin in China, we performed whole genome sequencing of *P. mirabilis* isolated from large-scale pig and chicken farms. Three new variants of PmGRI1 (HN31, YN8, and YN9), and a hybrid structure (HN2p) formed by the multidrug-resistant Tn*7*-like-HN2p transposon and a genomic island PmGRI1-HN2p, were identified from *P. mirabilis*. All variants underwent homologous recombination mediated by insertion sequence IS*26*. A genomic rearrangement in the chromosome between the Tn*7*-like-HN2p transposon and PmGRI1-HN2p occurred in HN2p. The heterozygous structure contained various antimicrobial resistance genes, including three copies of fluoroquinolone resistance gene *qnrA1* and 16S rRNA methylase gene *rmtB*, which are rarely found in *P. mirabilis*. Our results highlight the structural genetic diversity of genomic islands by characterizing the novel variants of PmGRI1 and enrich the research base of multidrug resistance genomic islands.

## 1. Introduction

*Proteus mirabilis* belongs to the *Enterobacteriaceae* family and is widely distributed in the environment and the intestinal tract of living organisms. It can carry numerous pathogenic factors that may be associated with gastrointestinal pathogenicity [1,2]. *P. mirabilis* is inherently resistant to antibiotics, such as nitrofurantoin, polymyxin, and tigecycline [3], often exhibiting multidrug resistance under clinical settings. Previous studies have reported multidrug resistance rates of 19.3–78.13% in *P. mirabilis* isolated from poultry [4,5,6]. Mobilizable genetic elements (genomic islands (GIs) and Tn*7*-like transposons) play important roles in the capture and transmission of multidrug resistance genes in *P. mirabilis* [7,8,9,10]. GIs, such as integrative and conjugative elements and integrative and mobilizable elements, can integrate gene fragments or single-stranded DNA into bacterial chromosomes via horizontal gene transfer and often contain various genes that confer novel traits to their hosts, such as antibiotic resistance, virulence, and enhanced adaptation of bacteria to their environment [11,12,13]. Recently, traces of GIs have been identified in an increasing number of host bacteria. Multidrug-resistant GIs carrying the macrolide resistance gene *erm*(B) have been reported in *Campylobacter* of animal origin, and *erm*(B) was found to be prevalent and significantly increased in *Campylobacter* in Guangdong Province, China. It is speculated that GIs may be widespread or play a role in the transmission of *erm*(B) [14]. A GI carrying a new variant of *tet*(L) was identified in *Campylobacter* of chicken origin; the variant was found to play an important role in tetracycline and doxycycline antibiotic resistance and could make tigecycline less susceptible [15]. GIs associated with glycopeptides, chloramphenicol, aminoglycosides, tetracyclines, sulfonamides, and β-lactam antibiotic resistance were identified in *Riemerella anatipestifer* by pan-genomic analysis [16]. Several novel multidrug-resistant GIs have been identified in *Trueperella pyogenes* of porcine origin, carrying multidrug resistance genes, such as the tetracycline resistance gene *tet*(W) and the macrolide resistance gene *erm*(X) [17]. Previous studies have reported that the most common genetic island in *P. mirabilis* is the *Salmonella* genomic island 1 (SGI1) [18,19,20] and the *Proteus* genomic island 1 (PGI1; [21,22]). The host bacteria range of SGI1 is very broad, and its presence in *P. mirabilis*, *Morganella morganii*, *Providencia stuartii*, and *Escherichia coli*, as reported in previous studies, suggests that SGI1 could spread to *Enterobacteriaceae* [18,23,24,25].

It has been reported that novel multidrug-resistant GIs, including PGI1, PGI2, and GIPmI1, were found in *P. mirabilis* [7,21,26]. PmGRI1 is a newly reported GI with variants ranging in size from 26,073 to 150,977 bp that can carry multiple resistance genes and has been identified in *P. mirabilis* and *E. coli*. It carries a tyrosine-type recombinant/integrase (394 amino acids) and is predicted to catalyze the integration of PmGRI1 at the 3′ end of tRNA(Sec). Different PmGRI1 variants with backbone alterations and/or variants in the MDR region carrying the carbapenemase gene *bla*_KPC-2_ and the 16S rRNA methylesterase gene *armA* have been identified in *P. mirabilis*, suggesting that PmGRI1 can carry clinically important resistance genes [27].

The Tn*7*-like transposon can transfer resistance genes [10,28]. Sequences at both ends of the Tn*7*-like transposon encode transposon modules and class 2 integrator systems. The transposon module encodes five proteins required for both transposition pathways: TnsA, TnsB, TnsC, TnsD, and TnsE [29]. Although these gene cassettes are fixed in Tn*7* transposons due to mutations in homologous recombinases, they can be rearranged in hosts expressing related recombinases, leading to alternative combinations of antibiotic resistance genes. Tn*7*-like transposons can promote the spread of resistance levels in bacteria by transferring various resistance genes between bacteria via transposases. Furthermore, a Tn*7*-like transposon, Tn*6450*, contains 18 different antimicrobial resistance genes, including the cephalosporinase *bla*_DHA-1_ and the fluoroquinolone resistance genes *qnrA1* and *aac(6****′****)-Ib-cr* [10].

## 2. Materials and Methods

### 2.1. Bacterial Strains

In 2019, 204 strains of *Proteus mirabilis* were isolated from the feces of large pig and chicken farms in Henan and Yunnan Provinces, China. The strains were cultured using Salmonella-Shigella agar (SS) at 37 °C for 16 h. The strains were identified using the BD Phoenix™ 100 Automated Microbiology System (Becton Dickinson, Franklin Lakes, NJ, USA).

### 2.2. Antimicrobial Susceptibility Testing

Antibiotic susceptibility testing of the strains was performed using the K-B diskdiffusion method. The 27th edition of the Executive Standard for Antimicrobial Susceptibility Testing (M100-S27), prepared by the Clinical and Laboratory Standards Institute, was used as a reference (Table S1). Drug sensitivity testing was carried out using Oxoid Paper Dispenser Type ST6090 and the OXOID drug sensitivity disks (OXOID, Basingstoke, UK). The quality control strain for the drug sensitivity test was *E. coli* ATCC25922. A total of 19 antibiotics were used for Antibiotic susceptibility testing. The antibiotics included ceftazidime (CAZ), aztreonam (ATM), levofloxacin (LEV), ampicillin (AMP), amoxicillin-clavulanic acid (AMC), cefoxitin (FOX), cefotaxime (CTX), chloramphenicol (CHL), imipenem (IPM), florfenicol (FFC), nalidixic acid (NAL), ciprofloxacin (CIP), streptomycin (STR), spectacularin (SPT), gentamicin (GEN), amikacin (AMK), methicillin (TMP), sulforaphane (SUL), and trimethoprim-sulfamethoxazole (SXT).

### 2.3. DNA Extraction

Bacterial genomic DNA was extracted using the QIAamp DNA Mini Stool kit (QIAamp, Hilden, Germany) according to the manufacturer’s instructions, and the genomic DNA concentration and quality were checked using a NanoDrop spectrophotometer and via agarose gel electrophoresis. The obtained DNA was stored at −20 °C until further analysis.

### 2.4. Whole Genome Sequencing and Analysis

Whole genome sequencing of 7 strains of multidrug-resistant *P. mirabilis* was selected from 204 strains of *P. mirabilis*. The whole genome of all strains was sequenced using the Illumina HiSeq platform (San Diego, CA, USA) (400 bp paired-end reads with about 200-fold average coverage) and a Nanopore sequencing instrument (MinION, Oxford, UK) (about 400-fold average read depth). After DNA extraction, 1 μg genomic DNA was randomly fragmented by Covaris (Woburn, MA, USA), followed by purification by an AxyPrep Mag PCR clean-up kit (Union City, CA, USA). The fragments were end-repaired by End Repair Mix and purified afterward. The repaired DNAs were combined with A-Tailing Mix, and then the Illumina adaptors were ligated to the Adenylate 3′Ends DNA and followed by product purification. The products were selected based on the insert size. Several rounds of PCR amplification with PCR Primer Cocktail and PCR Master Mix were performed to enrich the Adapter-ligated DNA fragments. After purification, the library was qualified by the Agilent Technologies 2100 (Palo Alto, CA, USA) bioanalyzer and ABI StepOnePlus Realtime PCR System (Foster City, CA, USA). Finally, the qualified libraries were sequenced pair-end using Hiseq System.

The raw sequencing data were processed using the following steps: (1) Removing reads containing sequencing adapter; (2) Removing reads whose low-quality base ratio (base quality less than or equal to 5) is more than 50%; (3) Removing reads whose unknown base (‘N’ base) ratio is more than 10%. Clean data were aligned to the reference genome using Burrows–Wheeler Aligner (BWA) [30]. Picard was used to remove duplicated sequence reads. Realignment was performed with the Genome Analysis Toolkit (GATK) [31]. Single-nucleotide polymorphisms (SNPs) and insertions-deletions (InDels) were called using HaplotypeCaller of GATK and annotated with SnpEff software [32]. The Copy Number Variants (CNVs) were called using the CNVnator read-depth algorithm [33]. CREST was used to identify structural variants (SVs) with standard settings [34].

The genomes were assembled using the Canu v1.5 software [35]. Antimicrobial resistance genes were identified using CGE ResFinder 3.1 (https://cge.cbs.dtu.dk/services/ResFinder/, accessed on 8 October 2021) [36]. Insertion sequences were identified using ISfinder (https://www-is.biotoul.fr/, accessed on 8 October 2021) [37]. The plasmids were identified using PlasmidFinder 2.1 [38,39]. The complete nucleotide sequence of the hybrid structure was analyzed using the BLAST program (http://blast.ncbi.nlm.nih.gov/Blast.cgi, accessed on 8 October 2021).

## 3. Results and Discussion

### 3.1. Antibiotic Susceptibility and Detection of Antimicrobial Resistance Genes

In addition to intrinsic resistance to doxycycline and polymyxin, *P. mirabilis* strains HN31, YN8, YN9, and HN2p are all insensitive to a variety of antibiotics and carry multiple drug resistance genes (Table 1). In addition, they all carry the merEDACPTR mercury-resistance operon. Our results highlight that PmGRI1 and the hybrid structure of Tn*7*-like and PmGRI1 can carry numerous antibiotic resistance genes, which may be one of the important reasons for the acquisition and spread of antibiotic resistance genes in *P. mirabilis*. The PlasmidFinder results showed that YN8 did not carry a plasmid and that HN31, YN9, and HN2P all carried IncQ1 plasmids but did not carry resistance genes on the plasmids. The resistance genes were relatively concentrated on PmGRI1.

### 3.2. Analysis of Three Novel Variants of PmGRI1 in P. mirabilis

**Figure 1:**
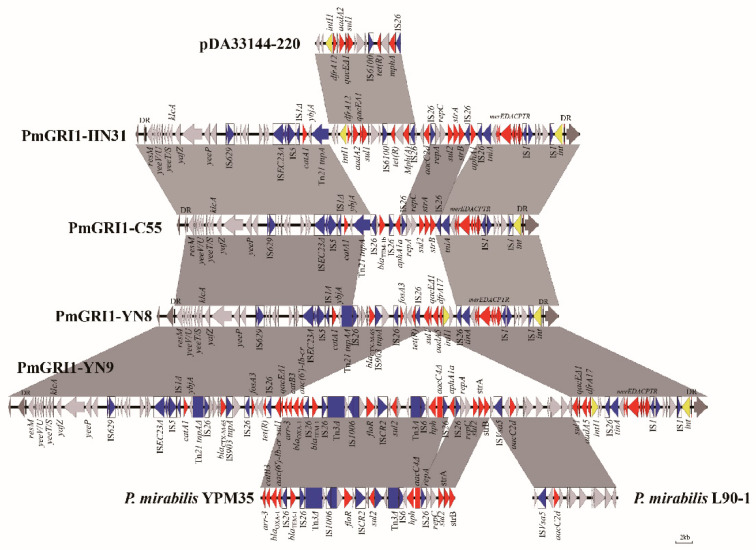
Genetic structure of three novel variants of PmGRI1. Structures were drawn to scale from NCBI accession numbers MW699442 (PmGRI1-HN31), MW699444 (PmGRI1-YN8), MW699445(PmGRI1-YN9), MK861851 (PmGRI1-C55), CP029591 (pDA33144-220), CP053898 (*P. mirabilis* YPM35), and CP045257 (*P. mirabilis* L90-1). Genes and open reading frames are indicated by arrows, and arrowheads indicate their orientations of transcription. Shared regions are indicated by shading with >99% identity. Antimicrobial resistance genes are shown in red, and transposase genes are shown in blue. The yellow arrow represents the integrase gene. The gray arrows represent other functional genes that have nothing to do with this research. The IS elements are indicated by boxes around the blue arrows. DR represents the 20 bp direct repeats at the ends of PmGRI1.

The whole genomes of strains HN31, YN8, and YN9 were compared by a BLAST search through NCBI, and all carried novel variants of PmGRI1 (PmGRI1-HN31, PmGRI1-YN8, and PmGRI1-YN9). The three novel variants of PmGRI1 were consistent with PmGRI1-C55 (*P. mirabilis* strain C55 was isolated from cloacal swabs of a chicken with diarrhea in Shandong Province, China, on 13 November 2018; NCBI GenBank MK861851.1, [27]) and were integrated downstream of tRNA(Sec) with 20 bp direct repeats (DR) at the left and right ends. They all underwent homologous recombination mediated by the insertion sequence IS*26*, due to which fragments from different plasmids or chromosomes were inserted, conferring new antibiotic resistance genes to the strain (Figure 1).

The size of PmGRI1-HN31 was 62,862 bp. The region downstream of Tn*21 tnpA* is similar to the fragment from *Klebsiella pneumoniae* plasmid pDA33144-220, both containing the resistance genes *dfrA12*, *aadA2*, *sul1*, and *mph*(A). This result suggests that this region may have been derived from pDA33144-220. Subsequently, insertions and reversals occur in the region between four IS*26*; an additional insertion of the aminoglycoside resistance gene *aacC2d* is upstream IS*26*, and the adjacent IS*26*-*bla*_TEM-1b_-IS*26*-*aphA1a* was reversed and transferred to a downstream IS*26*. However, IS*26* and *bla*_TEM-1b_ were lost in PmGRI1-HN31 compared to those in PmGRI1-C55.

PmGRI1-YN8 is 55,239 bp. The Tn*21 tnpA* of PmGRI1-YN8 was truncated by IS*26*, and a 14,852 bp sequence was inserted between the two IS*26*. This sequence carries five antibiotic resistance genes—*bla*_CTX-M-65_, *fosA3*, *sul1*, *aadA5*, and *dfrA17*.

PmGRI1-YN9 is 99,907 bp. It contains two sequences that are highly similar to *P. mirabilis* YPM35 and *P. mirabilis* L90-1 with a 99.9% nucleotide similarity. These two sequences carry the mobile elements IS*CR2* and IS*Vsa5*. The *sul1-qacEΔ1* gene cassette was observed on both ends of the inserted fragment, which may be associated with the insertion of a large fragment in this region. PmGRI1-YN9 contained 18 antibiotic resistance genes (Table 1).

IS*26* moves through a replication mechanism and can be used to cause insertion or deletion, or can flip adjacent DNA, playing an important role in pathogen evolution [40,41,42]. In addition, translocatable units containing only an IS*26* and a resistance gene are preferentially inserted into adjacent positions of the existing IS*26* in the same cell, resulting in an IS*26*-bounded class 1 transposon [43]. Previous studies have reported that IS*26*-mediated excision of the IS*26*-*aphA1a* gene transposon resulted in the loss of kanamycin resistance [44]. Our study showed that IS*26*-mediated homologous recombination of the backbone region and multidrug resistance region promoted the diversity of PmGRI1-like genomic island variants.

### 3.3. Characteristics of the Hybrid Structure in P. mirabilis

In the *P. mirabilis* strain HN2p, the SXT/R391 integrative and conjugative element ICEPmiJpn1 (GenBank accession KT894734), existing alone and harboring *bla*_CMY-2_, was integrated with the end of *prfC*. A resistant GI and a novel transposon derived from Tn*7* exist together and undergo genome rearrangement between the two elements.

**Figure 2:**
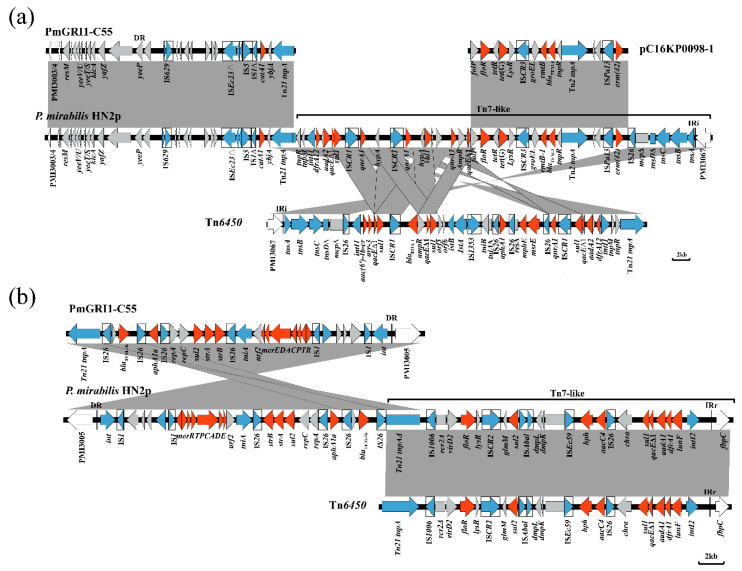
Genetic structures of the hybrid structure: (**a**) front end and (**b**) back end. Structures are drawn to scale from NCBI accession numbers MT585156 (hybrid structure in *P. mirabilis* HN2p), MK861851 (PmGRI1-C55), MF805806 (Tn6450), and CP052444 (pC16KP0098-1). Genes and ORFs are shown as arrows, and arrowheads indicate their transcription orientations. Shared regions with an identity of over 99% are indicated by shading. Antimicrobial resistance genes are in red, and transposase or integrase genes are in blue. The gray arrows represent other functional genes that have nothing to do with this research. The IS elements are indicated by boxes around the blue arrows. DR represents the 20-bp direct repeats at the ends of PmGRI1-C55. IRi and IRr are the inverted repeats that define the left and right ends of Tn6450, respectively.

The hybrid structure is formed by PmGRI1-HN2p and Tn*7*-like-HN2p (Figure 2). In HN2p, PmGRI1-HN2p is located between PMI3004 and PMI3005 compared with the *P. mirabilis* reference genome HI4320 (GenBank accession AM942759), and Tn7-like-HN2p is located at PMI3067. The two genetic elements have a Tn*21* region, one of which is incomplete, likely due to the truncation of IS*26* upstream. We hypothesized that an incomplete Tn*21* transposon mediates the transfer of the gene fragment and divides the assemblies into two parts (corresponding to bases 1537 to 76,562 bp and 148,476 to 197,071 bp in GenBank accession number MT585156). This indicates that the Tn*21* region might cause genome rearrangement. The region corresponding to PMI3005 to PMI3067 in *P. mirabilis* HI4320 was reversed between the two parts, and the complete inverse part had a length of 145 kb.

At the front end, PmGRI1-HN2p harbors the mobile element IS*629* and partial IS*Ec23* truncated by IS*5* and carries a *catA1* resistance gene followed by Tn*7*-like-HN2p, which might be derived from Tn*6450* by partial acquisition according to BLAST analysis (Figure 2a). The acquired part showed high sequence identity to the corresponding regions of four plasmids—pC16KP0098-1 (GenBank accession CP052444), pIncC-L117 (CP040034), pIncC-L121(CP040029), and pIncAC2_L111 (CP030132). The Tn*7*-like-HN2P region includes three copies of *qnrA1* and one copy of *rmtB*. Two of the three copies of *qnrA1* were downstream of IS*CR1*. The duplication of IS*CR1* around *qnrA1* might facilitate the enhancement of the *qnrA1* copy number and accompanying quinolone resistance under conditions of quinolone stress, and it likely adds to the repertoire of mechanisms that can improve quinolone resistance to clinically important levels. The presence of five copies of *qnrA1* was also reported in a previous study [45]. The presence of *rmtB* has been detected in *Escherichia coli*, *Klebsiella pneumoniae*, *Pseudomonas aeruginosa*, *Serratia marcescens*, and *P. mirabilis*, and the prevalence of such resistance determinants has become concerning [46,47,48,49,50,51]. The *rmtB* gene may be located between the Tn*2* transposon and insertion element [52,53]. In the Tn*7*-like-HN2p transposon, *rmtB* and *bla*_TEM-1_ coexist between the Tn*2* transposon and IS*CR3*.

At the back end (Figure 2b), a region similar to PmGRI1-C55 was inverted. An incomplete segment of Tn*2* and *bla*_TEM-1b_ is included between the two IS*26* and can confer resistance to penicillin and first-generation cephalosporins. IS*26* can promote the accumulation of resistance genes on gene islands. Excluding the Tn*21* region and five resistance genes (*bla*_TEM-1b_, *aphA1a*, *sul2*, *strA*, and *strB*), PmGRI1-HN2p carries a mercury-resistance operon. The 18,166-bp region of Tn*7*-like-HN2p (corresponding to bases 171,395 to 189,560 in MT585156), carrying *floR*, *sul2*, *aph(4)-Ia*, and *aacC4* resistance genes, showed high identity to the corresponding regions of seven IncHI2 plasmids—pHNSHP45-2 (GenBank accession KU341381), pHNYJC8 (KY019259), pWJ1 (KY924928), pHNLDF400 (KY019258), pHXY0908 (KM877269), pXGE1mcr (KY990887), and pSJ_255 (CP011062). Similar to previous reports, a class 1 integron was present in Tn*7*-like-HN2p containing *lun*(F), *dfrA1*, and *aadA1* cassettes [54].

The GI and Tn*7* transposons generate new variants through a series of molecular module rearrangements, acquisitions, or losses. The involvement of IS*3* and IS*21* elements in the rearrangement of multidrug resistance genes on GI has been reported in multidrug-resistant *E. coli* isolates [42]. In *Trueperella pyogenes* isolated from the lung tissue of cows in Jilin Province, China, multiple drug resistance genes were found to cluster on a 42 kb genomic island. Two IS*6100*Δ*1* and a class 1 integron-like SGI1 mediated genetic rearrangements and formed a complex transposon [55]. However, genome rearrangement occurring between the two genetic elements described in this study has seldom been reported. Inversion meditated by transposons and insertion elements found in bacteria might increase, and hybrid structures formed in this way might exist extensively in the natural environment. The effects it will cause and whether it will aggravate the dissemination of antimicrobial resistance genes require further research.

## 4. Conclusions

PmGRI1 is a novel GI that can carry multiple antibiotic resistance genes. The monitoring of PmGRI1 in *P. mirabilis* and other pathogenic bacteria is currently underway. In this study, we isolated and identified *P. mirabilis* from a large-scale farm using SS medium and the BD Phoenix™ 100 Automated Microbiology System and screened for multidrug-resistant *P. mirabilis* using the K-B disk diffusion method. We then performed whole-genome sequencing of the screened *P. mirabilis*. This study describes the detailed genetic structure of three novel variants of PmGRI1 and a hybrid structure in which the Tn*7*-like-HN2p transposon coexists with PmGRI1-HN2p in *P. mirabilis*. They all carry various important antibiotic resistance genes, which may lead to a more severe spread of antibiotic resistance genes in animals and the environment. This finding highlights the important role of genetic elements (Tn*7* and PmGRI1) in capturing and spreading resistance genes in *P. mirabilis*. Therefore, strong measures are required to control the emergence and spread of antibiotic resistance genes mediated by mobile genetic elements, and our study provides an important reference for this purpose.

## Figures and Tables

**Table 1:** Resistance genes and profile of PmGRI1 in *P. mirabilis*.

Strain	Source of Samples	SGI1/SGI1-like Gene Island Type	Antibiotic Resistance Genes ^a^	Strain Resistance Profile	Size (bp)
HN31	Swine	PmGRI1-HN31	***catA1***, ***dfrA12***, ***aadA2***, ***sul1***, ***mph(A)***, ***aacC2d***, ***sul2***, ***strA***, ***strB***, ***aphA1***, *floR*, *tet(A)*	AMP-CHL-NAL-STR-SPT-GEN-TMP-SUL-SXT	62,862
YN8	Chicken	PmGRI1-YN8	***catA1***, ***bla*_CTX-M-65_**, ***fosA3***, ***sul1***, ***aadA5***, ***dfrA17***, *aadA1*, *dfrA1*, *sul2*	NAL-SPT-TMP-SUL-SXT	62,784
YN9	Chicken	PmGRI1-YN9	***catA1***, ***bla*_CTX-M-65_**, ***fosA3***, ***sul1***, ***arr-3***, ***catB3***, ***bla*_OXA-1_**, ***aac(6′)-Ib-cr***, ***bla*_TEM-1_**, ***floR***, ***sul2***, ***hph***, ***aphA1a***, ***strA***, ***strB***, ***aacC2d***, ***aadA5***, ***dfrA17***, *aadA1*, *dfrA1*, *aacC4*	AMP-CHL-FFC-NAL-CIP-STR-SPT-GEN-TMP-SUL-SXT	55,239
HN2p	Swine	Hybrid structure of Tn7-like and PmGRI1	***rmtB***, ***hph***, ***aacC4***, ***aphA1a***, ***strA***, ***strB***, ***aadA1***, ***aadA2***, ***bla*_TEM-1b_**, ***qnrA1***, ***lun(F)***, ***erm(42)***, ***catA1***, ***floR***, ***sul1***, ***sul2***, ***tet(G)***, ***dfrA12***, ***dfrA1***, *bla*_CMY-2_	AMP-AMC-AMK-SXT-LEV-CIP-NAL-FFC-CHL-GEN	123,622
HN31	Swine	PmGRI1-HN31	***catA1***, ***dfrA12***, ***aadA2***, ***sul1***, ***mph(A)***, ***aacC2d***, ***sul2***, ***strA***, ***strB***, ***aphA1***, *floR*, *tet(A)*	AMP-CHL-NAL-STR-SPT-GEN-TMP-SUL-SXT	62,862
YN8	Chicken	PmGRI1-YN8	***catA1***, ***bla*_CTX-M-65_**, ***fosA3***, ***sul1***, ***aadA5***, ***dfrA17***, *aadA1*, *dfrA1*, *sul2*	NAL-SPT-TMP-SUL-SXT	62,784
YN9	Chicken	PmGRI1-YN9	***catA1***, ***bla*_CTX-M-65_**, ***fosA3***, ***sul1***, ***arr-3***, ***catB3***, ***bla*_OXA-1_**, ***aac(6′)-Ib-cr***, ***bla*_TEM-1_**, ***floR***, ***sul2***, ***hph***, ***aphA1a***, ***strA***, ***strB***, ***aacC2d***, ***aadA5***, ***dfrA17***, *aadA1*, *dfrA1*, *aacC4*	AMP-CHL-FFC-NAL-CIP-STR-SPT-GEN-TMP-SUL-SXT	55,239
HN2p	Swine	Hybrid structure of Tn7-like and PmGRI1	***rmtB***, ***hph***, ***aacC4***, ***aphA1a***, ***strA***, ***strB***, ***aadA1***, ***aadA2***, ***bla*_TEM-1b_**, ***qnrA1***, ***lun(F)***, ***erm(42)***, ***catA1***, ***floR***, ***sul1***, ***sul2***, ***tet(G)***, ***dfrA12***, *dfrA1*, *bla*_CMY-2_	AMP-AMC-AMK-SXT-LEV-CIP-NAL-FFC-CHL-GEN	123,622

^a^ Antimicrobial resistance genes carried by PmGRI1 or hybrid structure are indicated in bold.

## Data Availability

The datasets for this study were obtained from the NCBI MW699442 (PmGRI1-HN31), MW699444 (PmGRI1-YN8), MW699445 (PmGRI1-YN9), CP046048 (*P. mirabilis*-HN2p genome), and MT585156 (hybrid structure).

## Supplementary Materials

The following are available online at https://www.mdpi.com/article/10.3390/antibiotics10101268/s1, Table S1: Criteria for antibiotic susceptibility of *P. mirabilis*.
