# Supplementary material for: Identification of Three Novel PmGRI1 Genomic Resistance Islands and One Multidrug Resistant Hybrid Structure of Tn7-like Transposon and PmGRI1 in Proteus mirabilis
Source: Antibiotics (Basel). 2021 Oct 18;10(10):1268. doi: 10.3390/antibiotics10101268 (PMC8532799; doi:10.3390/antibiotics10101268)
Supplement: Supplementary file 1 [file antibiotics-10-01268-s001.zip › antibiotics-1412374-supplementary.pdf]

Supplementary Material

Supplementary Table

Table S1. Criteria for Antibiotic Susceptibility of *P. mirabilis*.

Diameter of antibacterial circle / antibiotics	antibiotics content (μg/Sheet)	Antibiotic resistance (mm)	Moderately sensitive (mm)	Sensitive (mm)
FFC	10	≤13	14-16	≥17
CAZ	30/20	≤20	/	≥21
ATM	30	≤17	18-20	≥21
LEV	5	≤13	14-17	≥18
NAL	30	≤13	14-18	≥19
C	30	≤12	13-17	≥18
AMP	10	≤13	14-16	≥17
AMC	20/10	≤13	14-17	≥18
FOX	30	≤14	15-17	≥18
CTX	30	≤22	23-25	≥26
IPM	10	≤19	20-22	≥23
FFC	30	≤14	14-18	≥19
CIP	5	≤15	16-20	≥21
SPT	100	≤10	11-13	≥14
CN	10	≤12	13-14	≥15
AK	30	≤14	15-16	≥17
TMP	5	≤10	11-15	≥16
SUL	300	≤12	13-16	≥17
SXT	25	≤10	11-15	≥16
S	30	≤13	14-17	≥18
